# Supplementary material for: Phytochrome-dependent responsiveness to root-derived cytokinins enables coordinated elongation responses to combined light and nitrate cues
Source: Nat Commun. 2024 Oct 1;15:8489. doi: 10.1038/s41467-024-52828-y (PMC11445486; doi:10.1038/s41467-024-52828-y)
Supplement: Supplementary file 3 — Description of additional supplementary files [file 41467_2024_52828_MOESM3_ESM.pdf]

# <u>Description of Additional Supplementary Files</u>

**Supplementary Data 1 -** Col0 HN cluster gene list

**Supplementary Data 2 -** Common targets ARRs Zubo 2017 Xie 2018
